# MRI-based intratumoral and peritumoral radiomics for preoperative prediction of glioma grade: a multicenter study

**DOI:** 10.3389/fonc.2024.1401977

**Published:** 2024-05-13

**Authors:** Rui Tan, Chunxiao Sui, Chao Wang, Tao Zhu

**Affiliations:** ^1^ Department of Neurosurgery, Tianjin Medical University General Hospital, Tianjin, China; ^2^ Department of Molecular Imaging and Nuclear Medicine, Tianjin Medical University Cancer Institute and Hospital, National Clinical Research Center for Cancer, Tianjin's Clinical Research Center for Cancer, Key Laboratory of Cancer Prevention and Therapy, Tianjin, China; ^3^ Department of Neurosurgery, Qilu Hospital of Shandong University Dezhou Hospital (Dezhou People’s Hospital), Shandong, China

**Keywords:** MRI, glioma grade, radiomics, peritumoral features, nomogram

## Abstract

**Background:**

Accurate preoperative prediction of glioma is crucial for developing individualized treatment decisions and assessing prognosis. In this study, we aimed to establish and evaluate the value of integrated models by incorporating the intratumoral and peritumoral features from conventional MRI and clinical characteristics in the prediction of glioma grade.

**Methods:**

A total of 213 glioma patients from two centers were included in the retrospective analysis, among which, 132 patients were classified as the training cohort and internal validation set, and the remaining 81 patients were zoned as the independent external testing cohort. A total of 7728 features were extracted from MRI sequences and various volumes of interest (VOIs). After feature selection, 30 radiomic models depended on five sets of machine learning classifiers, different MRI sequences, and four different combinations of predictive feature sources, including features from the intratumoral region only, features from the peritumoral edema region only, features from the fusion area including intratumoral and peritumoral edema region (VOI-fusion), and features from the intratumoral region with the addition of features from peritumoral edema region (feature-fusion), were established to select the optimal model. A nomogram based on the clinical parameter and optimal radiomic model was constructed for predicting glioma grade in clinical practice.

**Results:**

The intratumoral radiomic models based on contrast-enhanced T1-weighted and T2-flair sequences outperformed those based on a single MRI sequence. Moreover, the internal validation and independent external test underscored that the XGBoost machine learning classifier, incorporating features extracted from VOI-fusion, showed superior predictive efficiency in differentiating between low-grade gliomas (LGG) and high-grade gliomas (HGG), with an AUC of 0.805 in the external test. The radiomic models of VOI-fusion yielded higher prediction efficiency than those of feature-fusion. Additionally, the developed nomogram presented an optimal predictive efficacy with an AUC of 0.825 in the testing cohort.

**Conclusion:**

This study systematically investigated the effect of intratumoral and peritumoral radiomics to predict glioma grading with conventional MRI. The optimal model was the XGBoost classifier coupled radiomic model based on VOI-fusion. The radiomic models that depended on VOI-fusion outperformed those that depended on feature-fusion, suggesting that peritumoral features should be rationally utilized in radiomic studies.

## 1 Introduction

Glioma is a highly fatal disease that represents the most frequent form of primary cancer in the central nervous system (CNS), accounting for about 80% of all malignant tumors in the brain (1–3). Due to the same standardized treatment that can result in varying prognoses for different patients, it may be necessary to make specialized treatment decisions based on the tumor grade in clinical practice (4–6). Gliomas are categorized into grades I-IV in the Central Nervous System Midstream Classification of the World Health Organization (WHO) of 2021, with grades I-II being low-grade gliomas (LGG) and grades III-IV being high-grade gliomas (HGG) (7, 8). Accurate preoperative grading of gliomas is essential for assessing prognosis and developing individualized treatment plans, such as the extent of surgical resection, and the decision of postoperative chemoradiotherapy (9).

In contemporary clinical practice, gliomas are graded based on surgical or puncture histopathologic investigation (10). This diagnostic method is intrusive and slow, though. Furthermore, tissue biopsies from one area of the tumor might not be indicative of the histology of the entire tumor due to the recognized heterogeneity of gliomas and sampling error (11–13). High-precision noninvasive solutions that can offer preoperative grading information are therefore gaining popularity. Over the past decades, magnetic resonance imaging (MRI) has emerged as a crucial non-invasive diagnostic and assistant therapeutic technique for brain tumors, which is used to aid in differential diagnosis, guide treatment planning, and monitor therapy response (14–17). Nevertheless, competent radiologists may easily spot tumors from MRI sequences with the naked eye, gliomas are difficult to discriminate based on grade because of the variability and diversity of the tumors, which is undoubtedly a great challenge for imaging technology (18–20).

Radiomics, a burgeoning discipline, employing automated data mining algorithms to extract characteristics from medical images in a high-throughput manner, has been demonstrated notable advancements in the realm of medical imaging applications (21–24). These extracted features are then utilized by the machine to train itself and generate the anticipated desired output (25–27). Notably, recent progress has been achieved in the prediction of grading gliomas through the utilization of preoperative MRI scans and various machine learning methods. Gemini et al. evaluated the capacity of the Visually AcceSAble Rembrandt Images (VASARI) scoring system in predicting glioma grades and Isocitrate Dehydrogenase (IDH) status, with a possible application in machine learning (28). You et al. utilized traditional radiomics and the VASARI standard to construct a model determining glioma grade with an Area Under the Curve (AUC) of 0.966 (29). Wang et al. created and assessed a multiparametric MRI-based radiomics nomogram for predicting glioma grading (30). In recent years, deep learning has exhibited excellent performance with broader application prospects and deeper development in clinical applications. Voort et al. developed a single multi-task convolutional neural network that used preoperative MRI scans to predict the molecular subtype and grade of glioma, and the independent dataset evaluated that the approach achieved good performance and generalized well (31). Li et al. compared predictive models established by traditional radiomics and deep learning based on multiparametric MRI for grading gliomas and demonstrated that the latter performed better in most circumstances (32). While deep learning-based models have very respectable efficacy, it is commonly recognized as a black box that lacks satisfactory explanatory power. However, these machine learning approaches have focused mainly on the intratumoral region and neglected the role of the peritumoral environment in glioma grade.

The peritumoral environment holds great potential and may provide insightful information for clinical evaluation of the aggressive biological behavior of the tumor (33–35). Regarding intratumoral and peritumoral radiomic analysis, two main research approaches emerged. The first involved feature-fusion, where features from both intratumoral and peritumoral volumes of interest (VOIs) were separately extracted and then integrated. The second method was VOI-fusion, wherein the tumor region was expanded outward by a specific range to create a new VOI combining intra- and peri-tumoral areas. Radiomic features from this newly generated VOI were then extracted for subsequent analysis. For instance, Li et al. independently delineated the gross-tumor region and the peritumor region which was defined as the parenchyma that fell within a 2-cm distance to the tumor boundary (35). The radiomic features extracted from the two regions were merged and screened to build the radiomic model. Differently, Shi et al. expanded the originally segmented masks of VOIs by five radial distances outside the tumor at 1 mm intervals, creating five new VOIs (34). The findings indicated that the radiomic signature derived from peritumoral regions, specifically at dilated distances of 1 mm and 3 mm, demonstrated the most effective prediction performance in different MRI sequences, respectively. To date, although numerous studies have explored the radiomics of the peritumoral region, there has been a lack of a definitive study that has determined which among them is more persuasive and authoritative. The growth and infiltration of gliomas lead to the disruption of the blood-brain barrier. Consequently, there is a leakage of water, electrolytes, and proteins from peritumoral blood vessels, resulting in increased water content within the brain parenchyma, which contributes to the formation of peripheral edema (32, 36). Given that the tumor and the surrounding edematous area were closely interconnected, forming the microenvironment crucial for tumor cell growth and infiltration, it would be more reasonable to explore them as an integrated whole.

Drawing on the current state of research, the two methods were performed and compared in our study. We conducted an investigation focusing on the intratumor region and its surrounding peritumoral edema region of preoperative MRI scans to predict the grade of glioma. A total of 30 radiomic models, which depended on four different combinations of predictive features source (intratumoral VOI, peritumoral VOI, VOI-fusion, and feature-fusion), five different machine learning classifiers, and different MRI sequences, were established to select the optimal model, which was used for the construction of the nomogram for accurately predicting glioma grade, thereby assisting in the development of personalized treatment strategies for patients, ensuring they receive optimal benefits.

## 2 Materials and methods

### 2.1 Study population

All procedures involving human participants in this study adhered to the ethical guidelines outlined in the 1964 Declaration of Helsinki and its subsequent revisions, as well as other applicable ethical standards. The study was approved by the Ethics Committee of Tianjin Medical University General Hospital and Qilu Hospital of Shandong University Dezhou Hospital. Written informed consent was waived due to the retrospective nature of the study.

**Figure 1:**
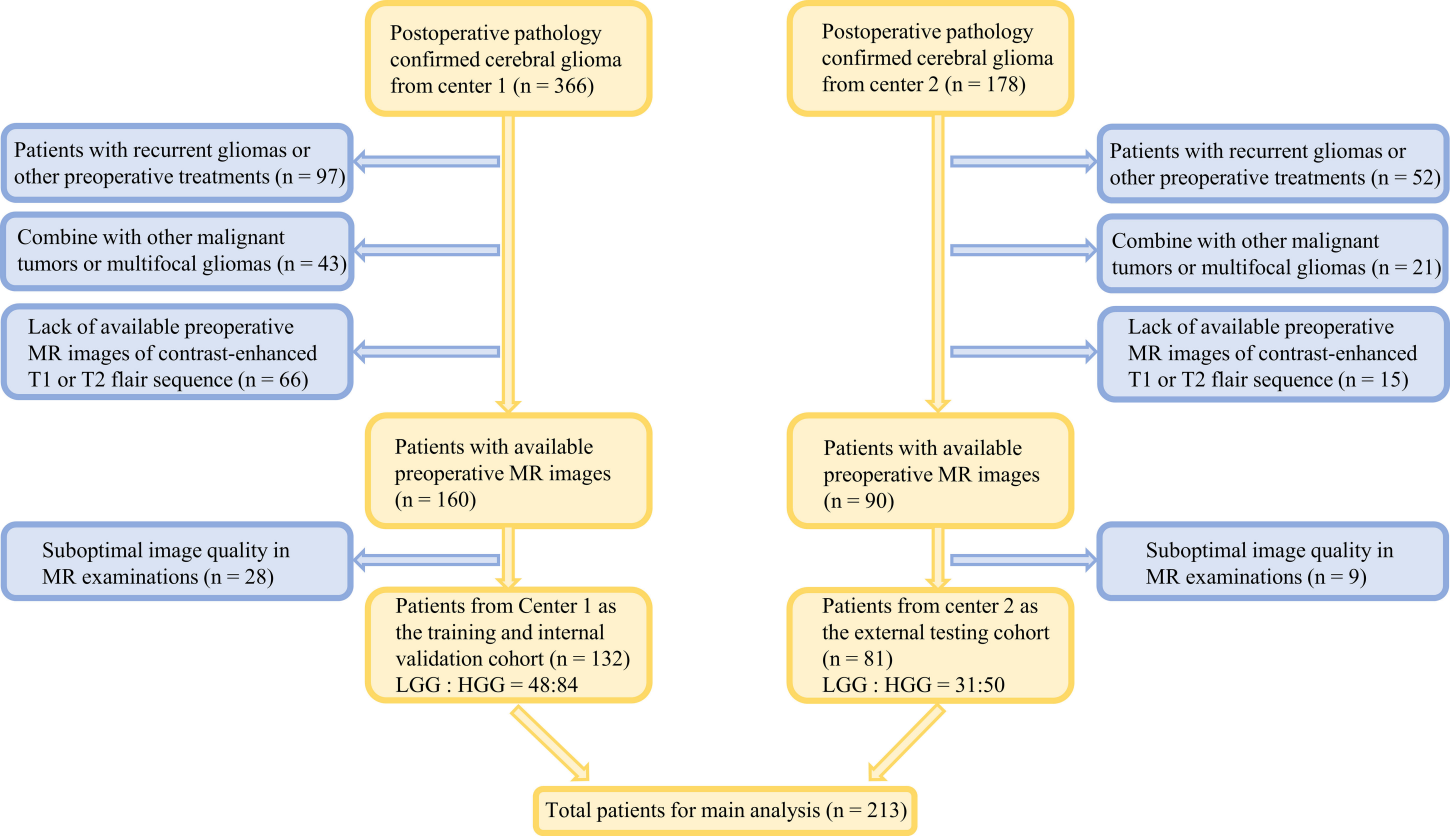
Flowchart of the incorporation and expulsion of glioma patients from two centers.

This study retrospectively analyzed 213 patients with cerebral gliomas from January 2019 to June 2023, who underwent preoperative MRI followed by surgery. Among them, 132 patients with glioma were from the Tianjin Medical University General Hospital (center 1), which was classified as the training cohort and internal validation set, and the remaining 81 patients were from the Qilu Hospital of Shandong University Dezhou Hospital (center 2), zoned as the independent external testing cohort. The inclusion and exclusion criteria are shown in **Figure 1**.

### 2.2 Pathological assessment

Pathologists with more than 10 years of experience graded the postoperative specimens, based on the 2021 WHO classification of CNS tumors, classifying gliomas into grades I-IV, with grades I-II being LGG and grades III-IV being HGG (19).

### 2.3 MRI protocol and image preprocessing

Every patient underwent MRI scans within two weeks before the surgery. All MRI studies were performed on the same type of scanners of the two centers, which were acquired using a 3.0 T scanner Discovery MR750 (GE Healthcare) with a 32-channel head coil. MRI acquisition parameters are summarized in **Supplementary Table S1**. The same MRI protocol was used for training and external testing sets. Patients were told not to move their heads during the scan to reduce the possible impact of head motion. The most useful anatomical multi-contrast MRI sequences included contrast-enhanced T1-weighted images and T2-weighted fluid-attenuated inversion recovery (Flair) images that were analyzed for further study.

For image preprocessing, the preoperative contrast-enhanced T1 images and T2 flair images were spatially aligned by using the rigid registration function of the well-validated Ants software from 3D Slicer (version: 5.2.2) software. Moreover, the quality of the registration was carefully inspected for alignment of the ventricular structures by a radiation oncologist. Then, the spacing of contrast-enhanced T1 and T2 flair images was resampled to 1×1×1 mm³.

### 2.4 Image segmentation and feature extraction

Manual segmentation of the contrast-enhanced T1 and T2 flair images of target lesions was performed using 3D Slicer (version: 5.2.2) software by two radiologists who possessed over five years of experience in a blinded manner to the study outcome, to eliminate unstable radiomic features and minimize inter-individual variability. Following clinical studies (8, 33), three VOIs have been delineated, the intratumoral VOI based on the contrast-enhanced T1 images, the peritumoral edema VOI including the intratumor VOI based on the T2 flair images, and the peritumoral edema VOI only. Given the spatial alignment between the contrast-enhanced T1 and T2 flair images, the delineated VOIs were shared across the two sequences.

In this study, the feature extraction was performed utilizing the Pyradiomics module in Python 3.7.0. A comprehensive set of 1288 quantitative radiomic features was extracted individually from each VOI for every sequence, obtaining a total of 7728 features. The available features were categorized as follows: the three-dimensional shape characteristics (n=14), the first-order statistical distribution of voxel intensities (n=252), and the texture features, which comprised gray-level co-occurrence matrix (GLCM) (n=308), gray-level dependence matrix (GLDM) (n=196), gray-level run length matrix (GLRLM) (n=224), gray level size zone matrix (GLSZM) (n=224), and neighborhood gray-tone difference matrix (NGTDM) (n=70).

### 2.5 Feature selection

Six distinct radiomic models were developed independently by employing extracted conventionally intratumoral radiomic features from two sequences and three kinds of VOIs. Firstly, the TR_T1_ model was a radiomic model based on contrast-enhanced T1-derived radiomic features from the intratumoral VOI. The TR_T2_ model was constructed by utilizing T2 flair-derived radiomic features from the intratumoral VOI. The TR model was established by integrating radiomic features originating from the intratumoral VOI of both sequences. Then, the PR model was based on the combined radiomic features from the peritumoral VOI of both sequences. The TPR_VOI-fusion_ model was constructed by utilizing the radiomic features from the combining VOI of intratumoral and peritumoral VOIs of both sequences. The TPR_feature-fusion_ model was established by integrating radiomic features from the intratumoral VOI and peritumoral VOI of both sequences, separately.

Subsequently, Z-score normalization was applied to standardize the intensity range of each radiomic feature across various models, preventing the undue assignment of lower or higher weights to specific features. In the feature selection process, three steps were implemented for the training cohort. The ICC test was conducted between the datasets obtained by the two radiologists. The value exceeding 0.75 was deemed indicative of robust reproducibility and reliability, leading to the exclusion of features with ICC<0.75 from subsequent analysis (37, 38). Furthermore, Pearson’s rank correlation coefficient was employed to evaluate the correlation between feature pairs, with one feature randomly excluded from each pair exhibiting a correlation coefficient > 0.9. Lastly, the least absolute shrinkage and selection operator (LASSO) regression, coupled with 10-fold cross-validation, was utilized to identify informative features with non-zero coefficients and calculate the corresponding feature weights.

In addition to radiomic features, machine learning models based on predictive clinical parameters were also constructed, which was referred to as the Clinical model in the study. For the feature selection of clinical features, a two-step procedure was performed. First, univariate analysis was used to identify significant features with a p-value < 0.05. Then, the stepwise multivariate analysis was employed to determine the independent indicator with a p-value < 0.05, which was utilized as the predictive clinical parameters for the prediction of glioma grade.

### 2.6 Model construction

Then, classifiers including Logistic Regression (LR), Support Vector Machine (SVM), eXtreme Gradient Boosting (XGBoost), Decision Tree (DT), and Multilayer Perceptron (MLP), employed the features filtrated by Lasso feature screening. In total, 30 machine learning models were formulated by integrating the six distinct radiomic models derived from various sequences and VOIs with the five machine learning classifiers for predicting glioma grade.

To ensure the stability of the prediction models, we randomly divided 30% of the training cohort as an internal validation set, then repeated the steps 100 times and averaged the results as the final prediction result under the model. The performance of the model was then evaluated on the independent external testing cohort, which was not used during the development of the model.

To evaluate the effectiveness of these models, several indicators, such as the AUC, accuracy, sensitivity, specificity, Positive Predictive Value (PPV), and Negative Predictive Value (NPV), were computed to evaluate the performance of the models. Additionally, to demonstrate the precision and net benefit, both the calibration curve and the decision curve analysis were employed.

Additionally, a nomogram using a logistic regression algorithm involving the optimal radiomic model and significant clinical features was developed to provide a straightforward visual representation in clinical practice. The receiver operating characteristic (ROC) curve, calibration curve, and decision curve analysis were employed correspondingly.

### 2.7 Statistical analysis

**Figure 2:**
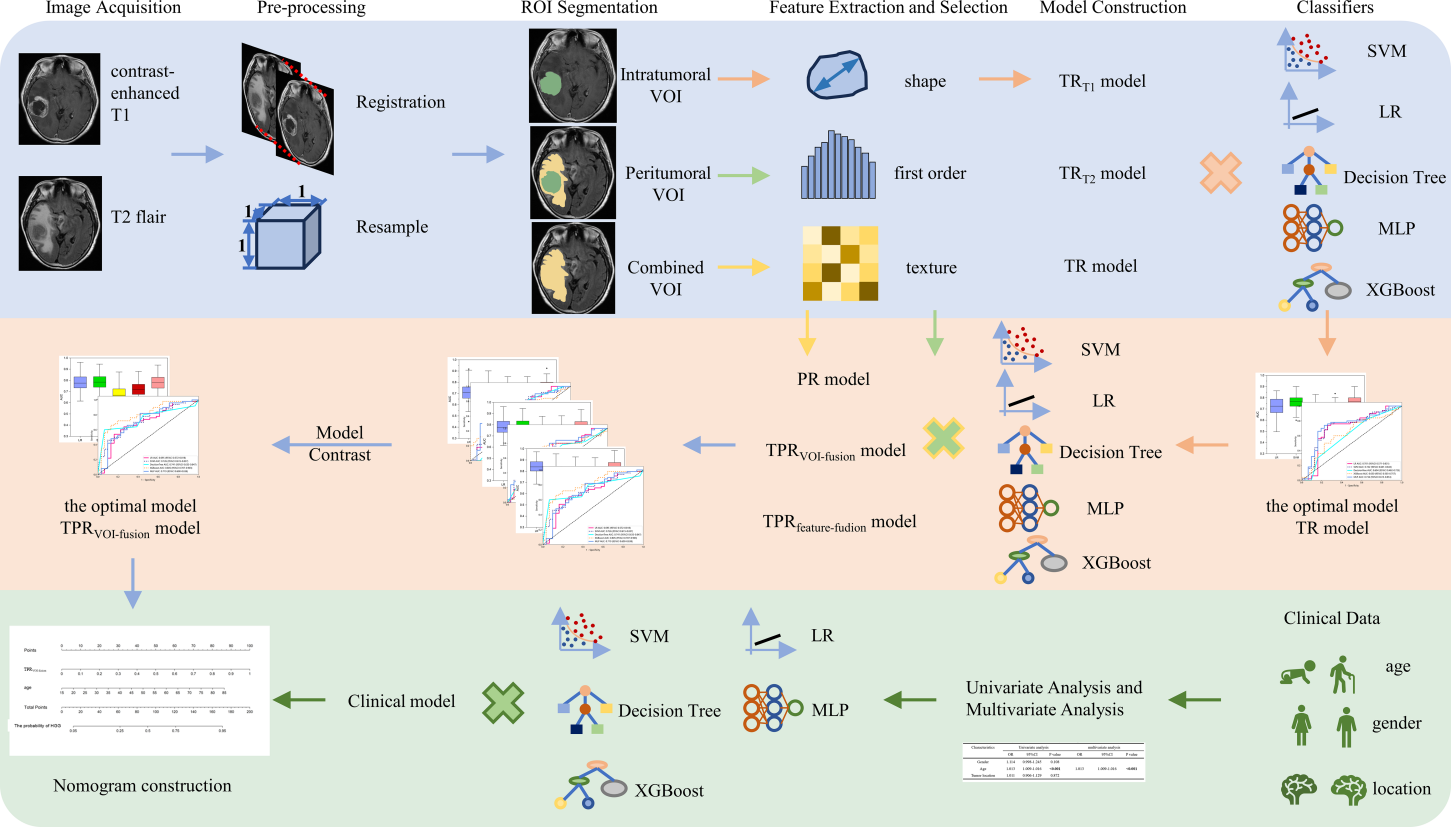
The workflow of intratumoral and peritumoral radiomics and clinical analysis for image acquisition, segmentation, feature extraction and selection, model comparison, and nomogram construction in our study.

Software version 3.7.0 of Python was used for statistical analysis. The p-value for statistical significance was fixed at 0.05, and all statistical tests were two-sided. For continuous variables, mean ± SD was applied to communicate data that followed a normal distribution; and counts and percentages (n, %) were utilized to convey data for categorical variables. To compare continuous and categorical variables, t-tests and Chi-square were wielded. The prediction result of each model was displayed on an ROC curve, and the prediction performance was evaluated by calculating the AUC, accuracy, sensitivity, specificity, PPV, and NPV. The Delong test was utilized to verify the significance of the AUC from different ROC curves. The Hosmer-Lemeshow test was employed to evaluate the fitting ability of the model. The entire workflow of this study is illustrated in **Figure 2**.

## 3 Results

### 3.1 Patient characteristics

A total of 213 glioma patients fulfilled the requirements for admission from the two centers, 132 patients from center 1 were classified as the training cohort and internal validation set (84HGGs, 48LGGs, mean age 52 years), and the remaining 81 patients from center 2 were zoned as the independent external testing cohort (50HGGs, 31LGGs, mean age 52 years). **Table 1** displays the variations in the clinical features of the two groups from different centers. It can be shown that the only factor that significantly distinguished HGG from LGG was age (p<0.001). The gender and tumor location were not proposed as potential predictors of glioma grade.

**Table 1:** Demographic information and clinical characteristics of glioma patients.

Characteristics	Center 1 LGG (n=48)	Center 1 HGG (n=84)	P value		Center 2 LGG (n=31)	Center 2 HGG (n=50)	P value
Gender			0.493				0.21
Male	26 (54.17%)	52 (61.90%)			14 (45.16%)	31 (62.00%)	
Female	22 (45.83%)	32 (38.10%)			17 (54.84%)	19 (38.00%)	
Age	44.81±14.73	56.31±14.24	**<0.001** ^*^		44.00±11.57	56.26±13.11	**<0.001** ^*^
Tumor location			0.755				0.876
Right brain	25 (52.08%)	40 (47.62%)			16 (51.61%)	28 (56.00%)	
Left brain	23 (47.92%)	44 (52.38%)			15 (48.39%)	22 (44.00%)	

A t-test was used for age. A χ^2^ test was used for the rest. ^*^p<0.05

LGG, low-grade glioma; HGG, high-grade glioma.

Significant p values (p< 0.05) are indicated in bold.

### 3.2 Machine learning models based on intratumoral radiomics

For intratumoral radiomics, a total of 2576 (1288 contrast-enhanced T1-based and 1288 T2 flair-based radiomic features) features were extracted from each tumor region, containing shape, first-order, and texture features. Hence the TR_T1_, TR_T2_, and TR models included 1288, 1288, and 2576 radiomic features respectively. The feature statistics of categories and distribution are presented in **Supplementary Figure S1**. After feature filtration of the ICC test and Pearson’s rank correlation coefficient, the LASSO with 10-fold cross-validation was employed to select significant features for building radiomic signatures. At last, 7 features were selected for the TR_T1_ model (**Supplementary Figure S2A**), 7 features were selected for the TR_T2_ model (**Supplementary Figure S2B**), and 6 features were selected for the TR model (**Supplementary Figure S2C**), with weighting coefficient severally. The coefficients and mean standard error (MSE) of the 10-fold validation are also exhibited in **Supplementary Figure S2**.

**Figure 3:**
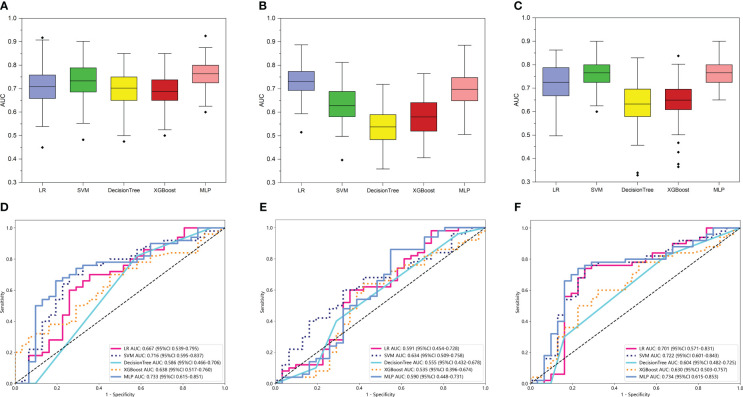
The performance of intratumoral radiomic models based on the different MRI sequences. The performance of the internal validation employed 30% of the training cohort randomly, and repeated the steps 100 times of TR_T1_ **(A)**, TR_T2_ **(B)**, and TR **(C)** models respectively. Comparison of ROC curves for the external test of TR_T1_ **(D)**, TR_T2_ **(E)**, and TR **(F)** models of five machine learning models. The models that the mean AUC of the internal validation throughout the 100 repetitions reached more than 0.7, were considered to be relatively stable and efficient. Comprehensively, the MLP-based TR model (AUC = 0.734 in external test) based on the two MRI sequences was considered the optimal model and selected for further analysis.

Then, 15 machine learning models were constructed based on the various radiomic models above-mentioned and machine learning classifiers (LR, SVM, XGBoost, DT, and MLP) to determine which model was optimal for glioma grade prediction. The results of internal validation with randomized division repeated 100 times are shown in **Figures 3A–C** for the TR_T1_, TR_T2_, and TR models in sequence. Among these five categories of machine learning models, the MLP-based TR_T1_ model, LR-based TR_T2_ model, and MLP-based TR model, which had the highest mean values of 0.76, 0.73, and 0.77, respectively, were considered the most stable models. In the external testing set, the performance of each category classifier on the TR_T1_ and TR model outperformed that of the TR_T2_ model respectively (**Figure 3E**, **Supplementary Table S2**). Ultimately, the MLP-based TR model (**Figure 3F**) with an AUC of 0.734 and MLP-based TR_T1_ model (**Figure 3D**) with an AUC of 0.733, were considered the optimal model in intratumoral radiomics. Although the Delong test showed a non-significantly statistic (*p* = 0.966) between these two models, the accuracy, sensitivity, and specificity of the MLP-based TR model were higher than the MLP-based TR_T1_ model. Hence, the TR model based on double-sequence MRI was chosen for further research and evaluation. The decision curves and calibration curves are also depicted in **Supplementary Figure S4**.

### 3.3 Machine learning models based on peritumoral radiomics

**Figure 4:**
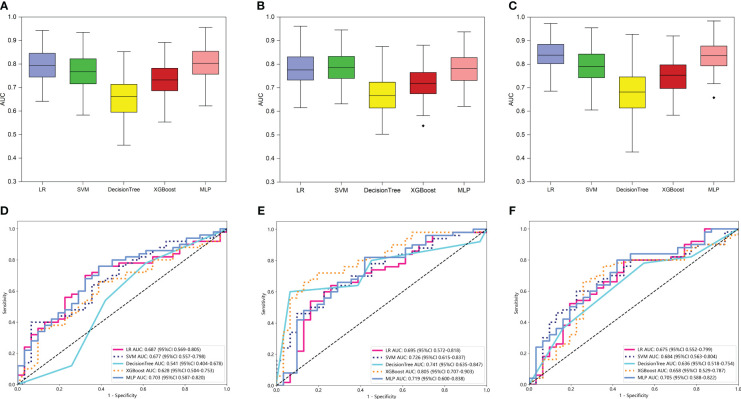
The performance of intratumoral and peritumoral radiomic models based on the two MRI sequences. The performance of the internal validation employed 30% of the training cohort randomly, and repeated the steps 100 times of PR **(A)**, TPR_VOI-fusion_ **(B)**, and TPR_feature-fusion_ **(C)** models respectively. Comparison of ROC curves for the external test of PR **(D)**, TPR_VOI-fusion_ **(E)**, and TPR_feature-fusion_ **(F)** models of five machine learning models. Ultimately, the XGBoost-based TPR_VOI-fusion_ model (AUC = 0.805 in the external test) was identified as the best model to develop a nomogram.

For peritumoral radiomics, the PR models contained 2576 radiomic features (**Supplementary Figure S1**). After feature selection, 8 features were finally selected for the PR model (**Supplementary Figure S3A**). **Figure 4A** shows the performance of 100-repetition randomized division internal validation. In the external testing cohort, only the AUC of the MLP-based PR model exceeded 0.7, indicating a lower predictive performance compared to the intratumoral radiomics (**Figure 4D**).

### 3.4 Machine learning models based on intra- and peri-tumoral fusion radiomics

For intra- and peri-tumoral fusion radiomics, the TPR_VOI-fusion_ models contained 2576 radiomic features, while the TPR_feature-fusion_ model included 5152 (**Supplementary Figure S1**), due to the different combinations of feature sources. After radiomic features dimensionality reduction, the LASSO regression finally selected 6 features for the TPR_VOI-fusion_ model (**Supplementary Figure S3B**), and 9 features for the TPR_feature-fusion_ model (**Supplementary Figure S3C**). Analogically, the two radiomic models were combined with 5 kinds of classifiers previously mentioned to develop machine-learning models for predicting glioma grade. The 100-repetition randomized division internal validation was conducted to evaluate the model’s performance stability. Except for the DT model, the mean AUC of the other four machine learning models reached more than 0.7 in internal validation, which were considered to be relatively stable and efficient predicting models (**Figures 4B, C**). In the external testing, each categorical classifier on the TPR_VOI-fusion_ model outperformed that of the TPR_feature-fusion_ model respectively (**Table 2**, **Figures 4E, F**). The XGBoost-based TPR_VOI-fusion_ model with an AUC of 0.805 was considered the optimal model (**Figure 4E**) in all intratumoral and/or peritumoral radiomic models. **Supplementary Figure S5** exhibits the decision curves and calibration curves of corresponding models.

**Table 2:** Each evaluation index of PR, TPR_VOI-fusion_, and TPR_feature-fusion_ models of five machine learning classifiers in the external test.

Model	Classifier	AUC	95%CI	Accuracy	Sensitivity	Specificity	PPV	NPV
PR	LR	0.687	0.5689-0.8053	0.667	0.760	0.516	0.717	0.571
	SVM	0.677	0.5570-0.7978	0.654	0.820	0.387	0.683	0.571
	DT	0.541	0.4037-0.6776	0.556	0.540	0.581	0.675	0.439
	XGBoost	0.628	0.5038-0.7529	0.630	0.680	0.548	0.708	0.515
	MLP	0.703	0.5867-0.8197	0.667	0.840	0.387	0.689	0.600
TPR _VOI-fusion_	LR	0.695	0.5718-0.8178	0.654	0.820	0.387	0.683	0.571
	SVM	0.726	0.6147-0.8370	0.654	0.840	0.355	0.677	0.579
	DT	0.741	0.6352-0.8474	0.704	0.800	0.548	0.741	0.630
	XGBoost	0.805	0.7069-0.9034	0.691	0.820	0.484	0.719	0.625
	MLP	0.719	0.6002-0.8385	0.667	0.840	0.387	0.689	0.600
TPR _feature-fusion_	LR	0.675	0.5516-0.7993	0.704	0.800	0.548	0.741	0.630
	SVM	0.684	0.5634-0.8044	0.691	0.800	0.516	0.727	0.615
	DT	0.636	0.5181-0.7536	0.654	0.780	0.452	0.696	0.560
	XGBoost	0.658	0.5293-0.7868	0.667	0.800	0.452	0.702	0.583
	MLP	0.705	0.5880-0.8224	0.667	0.840	0.387	0.689	0.600

AUC, area under the curve; CI, confidence interval; PPV, positive predictive value; NPV, negative predictive value.

### 3.5 Nomogram construction

The predictive clinical parameters were chosen based on univariate and multivariate analyses. As shown in **Table 3**, age was found to be an independent predictor (OR 1.013; 95% CI 1.009-1.016; p<0.001) for glioma grade prediction. Then, the Clinical models were built based on the selected independent predictor and 5 kinds of classifiers.

**Table 3:** Univariate analysis and multivariate analysis of clinical characteristics in all patients.

Characteristics	Univariate analysis			multivariate analysis		
	OR	95%CI	P value	OR	95%CI	P value
Gender	1.114	0.998-1.245	0.108			
Age	1.013	1.009-1.016	**<0.001** ^*^	1.013	1.009-1.016	**<0.001** ^*^
Tumor location	1.011	0.906-1.129	0.872			

OR, odds ratio; CI, confidence interval.

^*^p<0.05.

Significant p values (p< 0.05) are indicated in bold.

**Figure 5:**
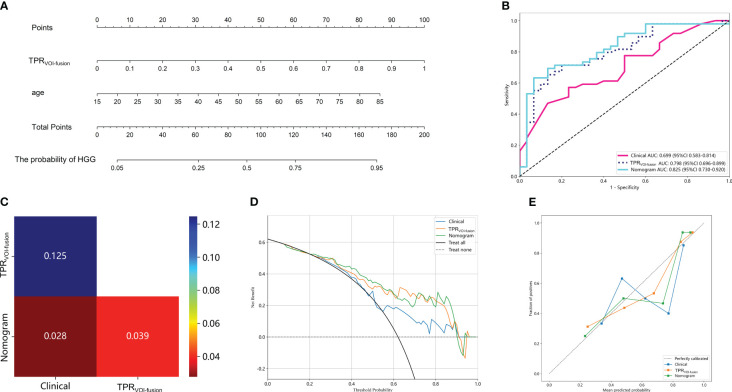
Clinical application of the nomogram constructed by radiomic model and clinical parameter in predicting the probability of being HGG for glioma patients **(A)**. The total point was obtained by adding the scores located on the TPR_VOI-fusion_ and age coordinate axis together, and the vertically corresponding value on the bottom line was the probability of being HGG. **(B)** Comparison of ROC curves of the Clinical model, TPR_VOI-fusion_ radiomic model, and nomogram. **(C)** The Delong test among the three models. The nomogram with an AUC of 0.825 in the test cohort, significantly outperformed the other two models. The DCA **(D)** and calibration curves **(E)** of the three models. The nomogram had a higher net benefit in predicting glioma grade and represented an ideal fit.

Subsequently, in exploring the potential utility of the developed MRI-based intratumoral and peritumoral radiomic models for preoperative prediction of glioma grade, a nomogram was constructed by combining the clinical independent predictor with the optimal TPR_VOI-fusion_ machine learning model (**Figure 5A**). The individual risk of being predicted as an HGG glioma was derived from the cumulative total points obtained, which allowed for the representation of the prediction model in a more simplified and comprehensive manner. **Figure 5B** illustrates the superior performance of the nomogram with an AUC of 0.825 (testing cohort), in comparison to both the Clinical model and TPR_VOI-fusion_ radiomic model. After the Delong test, the nomogram was proved to significantly outperform both of the models (**Figure 5C**), and the net benefit in the DCA curve of the nomogram was higher than that of the two models at threshold probabilities in the testing cohort (**Figure 5D**). The Hosmer-Lemeshow test revealed favorable calibration of the nomogram (*p* = 0.089), suggesting alignment with an ideal fit without significant deviation (**Figure 5E**).

## 4 Discussion

In this study, we developed variously dependable models to preoperatively predict grade glioma by using MRI images, which were constructed by radiomic features extracted from the intratumoral region and peritumoral edema region. The XGBoost machine learning classifier, incorporating features extracted from the combination of intratumoral and peritumoral VOIs of MRI, exhibited superior performance in distinguishing between LGG and HGG. The internal validation and independent external test underscored the robustness and generalizability of the model. Additionally, the radiomic models derived from VOI-fusion outperformed those derived from feature-fusion in our study, suggesting more extensive investigations into peritumoral radiomics were necessary to determine a more standardized research method and provide more theoretical support for radiomic studies. Finally, a nomogram based on the optimal machine learning model and clinical parameter was established to detect potential applications for predicting glioma grade in clinical practice.

MRI serves as a routine tool for preliminary diagnosing, treatment planning, and monitoring the treatment response of patients with glioma (39, 40). Recent studies have consistently demonstrated a robust association between radiomic features extracted from multiparametric MRI scans and various applications related to gliomas (2, 41). Kim et al. distinguished between glioblastoma and primary CNS lymphoma using multiparametric MRI sequences that included contrast-enhanced T1-weighted, T2-weighted, and diffusion-weighted imaging (42). Fifteen features were chosen from all imaging modalities, and the model rendered an AUC of 0.979, demonstrating the potent prediction potential of multi-parameter MRI. Nevertheless, the prediction of models based on a single MRI sequence was not performed and compared. In our study, we constructed and compared various radiomic models based on the intratumoral region of the single contrast-enhanced T1 sequence, the single T2 flair sequence, and the combination of two sequences. As expected, the radiomic models derived from dual-sequence MR imaging outperformed those solely based on single contrast-enhanced T1 or single T2 flair sequence. Hence, multicontrast MRI-based radiomics was poised to enhance the predictive capability for glioma grading compared to that of single MRI sequence.

Previous studies have demonstrated that the heterogeneity of gliomas extended beyond the tumor interior to include the peritumoral region, where approximately 90% of gliomas recurred (43, 44). Glioma cells interacted with molecules in the peritumoral area to cause hypoxia, angiogenesis, and tumor infiltration, which would ultimately accelerate the growth of gliomas (45). Consequently, the significant potential existed in the peritumoral environment, which could provide important information for evaluating the aggressive biological behavior of the tumor clinically (46, 47). In studies of other cancers, Ding et al. investigated the effect of peritumoral features for predicting sentinel lymph node metastasis in breast cancer (48). They created peritumoral regions by expanding tumor regions of interest at thicknesses of 2 mm, 4 mm, 6 mm, and 8 mm. By incorporating peritumoral features, the accuracy in the validation set increased from 0.704 to 0.796. Shan et al. developed a prediction model using peritumoral radiomic signatures extracted from a 2 cm peritumoral area, assessing its effectiveness in predicting the early recurrence of hepatocellular carcinoma post-curative treatment (49). In the validation cohort, ROC curves and decision curves revealed superior prediction efficiency and greater clinical benefits with the peritumoral model. Consequently, the extraction and integration of peritumoral and intratumoral features present a promising avenue. However, existing radiomic-based techniques for grading gliomas focused primarily on the interior of the tumor and less on the peritumoral environment.

Peripheral edema and peritumor in gliomas are two distinct concepts, the peritumor is typically an area within a specified radius around the tumor, whereas the peripheral edema around a glioma is irregular and frequently dispersed along the cerebral gyrus. Previous research has shown that the degree of peritumoral edema increases with the pathological grade and aggressiveness of glioma (14). It was demonstrated that individuals with severe edema (>10 mm) had mean or overall survival rates that were more than 50% lower than those with mild edema (8, 9, 44). Cheng et al. compared the predictive power of the peritumor and peripheral edema region for grading gliomas and found the most predictive features were extracted from the peritumor region within an immediate distance of 1 mm from the tumor core based on MRI scans (8). Notably, the study did not explore a predictive model based on the peripheral edema region in combination with the intratumoral region. Considering that the prognosis of gliomas was strongly correlated with the occurrence of peritumoral edema, we attempted to investigate the radiomic models based on peripheral edema for glioma grade.

Concerning the two main research approaches in intratumoral and peritumoral radiomic analysis, which was simplified as feature-fusion and VOI-fusion, definitive studies remained absent establishing a persuasive conclusion about which approach made more sense and produced more predictive features. Our study filled this gap by conducting a comparative analysis of the two methodologies for the first time. In the internal validation, the AUC of models based on both two methods showed no obvious differences, the difference in AUC was consistent across classifiers. In the external test, each categorical classifier on the VOI-fusion model outperformed that of the feature-fusion model respectively, which indicated that the model constructed by extracting features from the intratumoral and peritumoral regions as a whole yielded higher prediction efficiency. Regarding VOI fusion, the radiomic features, such as shape, first-order, and texture, extracted comprehensive information taking into account both intratumoral and peripheral edema region. Regarding feature fusion, individual peritumoral features exhibited minimal statistical variance in effectively distinguishing HGG from LGG, rendering them prone to elimination during screening. In our study, the constructed model just by feature fusion performed poorly in external tests, indicating unstable performance. This highlighted the need for further studies to unravel the intricacies of intratumoral and peritumoral radiomics.

We assessed 1288 radiomics features for every MRI sequence, and 7728 features in total, which was distinctly more than most recent findings and included all significant variables for radiomic analysis (30, 45). To identify the most optimal model for our dataset, we applied the six previously discussed categories of radiomic models across five classifiers: LR, SVM, XGBoost, DT, and MLP. This meticulous process ensured the exploration of fully optimized models best suited for our data. Meanwhile, previous studies have identified several clinical parameters crucial in distinguishing between LGG and HGG. Wang et al. selected clinical factors including age and sex as well as radiomic signature to develop a nomogram to predict glioma grading (30). It is well-recognized that HGG tends to be diagnosed in the elderly (31, 50). Consistently, we found that only the age parameter was statistically significant in predicting glioma grading, using the uni-multivariate analyses. Despite the lower predictive capacity of clinical model based solely on age feature compared to radiomic models, the nomogram amalgamating clinical parameter and radiomic models surpassed the predictive efficacy of either model in isolation. In terms of the predictive efficiency of various machine learning methods, Voort et al. utilized a deep learning model to predict the grade of glioma, achieving an AUC of 0.81 in the external testing cohort of 240 patients from 13 different institutes (31). Similarly, Li et al. distinguished LGG from HGG by developing deep convolutional neural network models, achieving an AUC of 0.89. In comparison, the nomogram in our study showed great performance with an AUC of 0.825 in the independent external testing cohort, which was equivalent to the state-of-the-art research aforementioned.

For all this, there were limitations in this study. First, our study required a larger sample size from more centers to make the findings more convincing. Second, only two MRI sequences were employed in this study. Some advanced parametric MRI scans, such as DWI and DTI, have shown powerful potential in tumor research, and new scanning techniques should be explored (7, 18, 36). Third, the VOIs in our study were manually annotated, which was time-consuming and laborious, and even prone to inaccurate annotation. Deep learning-based tumor segmentation methods are expected to be employed to improve the accuracy and reliability of image segmentation. Finally, our study lacked molecular subtyping of the samples, which was critical for the prognosis of gliomas, and planned to integrate such information in future studies.

## 5 Conclusion

In this work, we assessed the function of radiomic models of intratumoral and peripheral edema regions in MRI scans for predicting glioma grade and validated the methodology on an independent external test dataset, which provided a fresh viewpoint on the disease. The nomogram combined clinical parameter and the optimal radiomic model was efficient in glioma grade, and this non-invasive approach was expected to promote clinical research and guide the management of individualized glioma treatment.

## Data availability statement

The raw data supporting the conclusions of this article will be made available by the authors, without undue reservation.

## Ethics statement

The studies involving humans were approved by Tianjin Medical University General Hospital’s Institutional Ethics Committee. The studies were conducted in accordance with the local legislation and institutional requirements. The ethics committee/institutional review board waived the requirement of written informed consent for participation from the participants or the participants’ legal guardians/next of kin because The requirement for written informed consent forms from all patients taking part in the study was waived because of the retrospective investigation.

## Author contributions

RT: Data curation, Writing – original draft. CS: Methodology, Writing – original draft. CW: Writing – original draft. TZ: Conceptualization, Writing – review & editing.
