# Supplementary material for: MRI-based intratumoral and peritumoral radiomics for preoperative prediction of glioma grade: a multicenter study
Source: Front Oncol. 2024 May 13;14:1401977. doi: 10.3389/fonc.2024.1401977 (PMC11128562; doi:10.3389/fonc.2024.1401977)
Supplement: Supplementary file 1 [file DataSheet_1.docx]

**Supplementary Material**

MRI-Based Intratumoral and Peritumoral Radiomics for Preoperative Prediction of Glioma Grade: a multicenter study

**Figure**


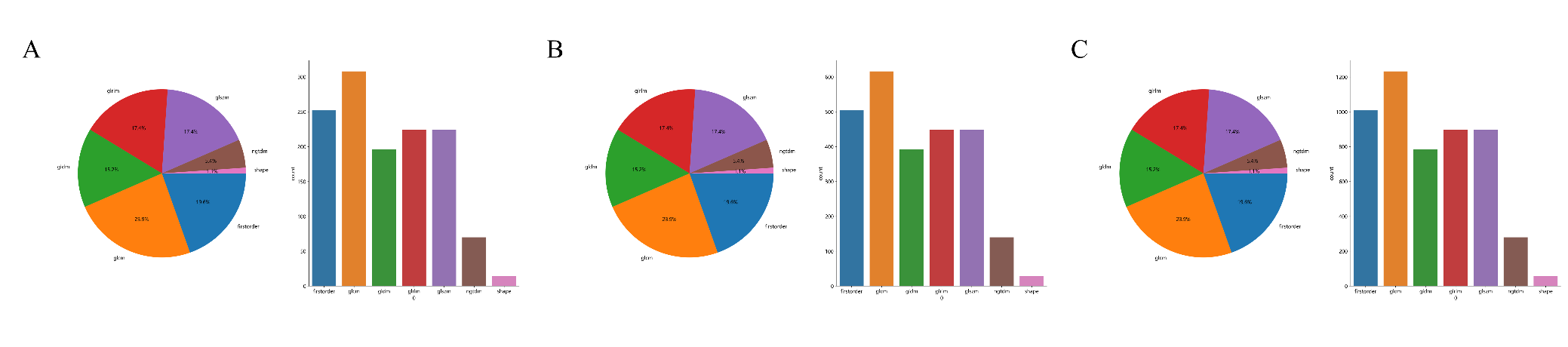


**Figure S1.** The feature statistics of categories and distribution of the TR_T1_ model and TR_T2_ model (A); the TR model, PR model, and TPR_VOI-fusion_ model (B); and the TPR_feature-fusion_ model (C).

**
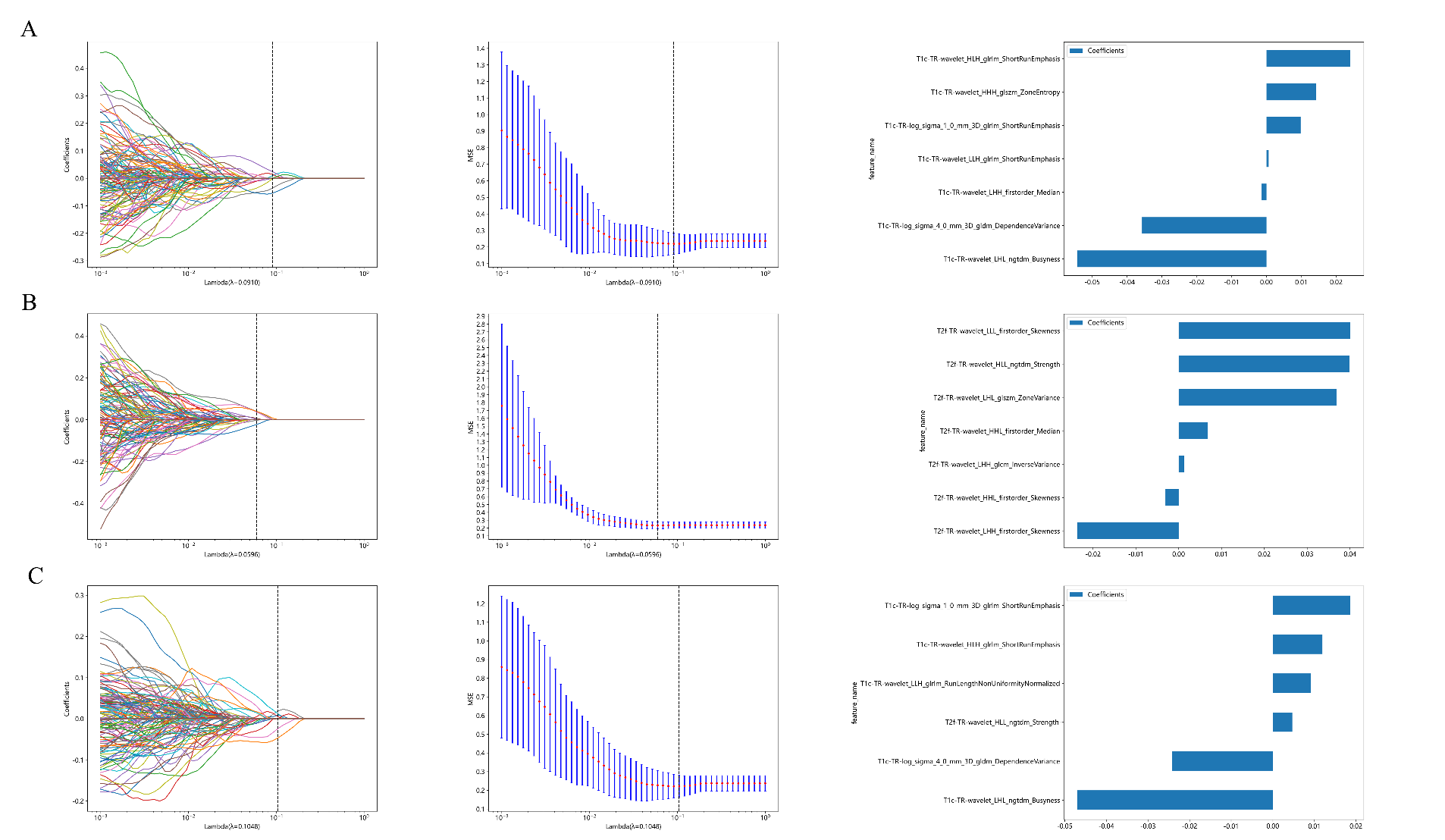
**

**Figure S2.** The intratumoral radiomic features dimension reduction by using a LASSO algorithm. The coefficients and MSE of 10-fold cross-validation and the histogram of coefficients of TR_T1_ (A), TR_T2_ (B), and TR (C) models respectively.


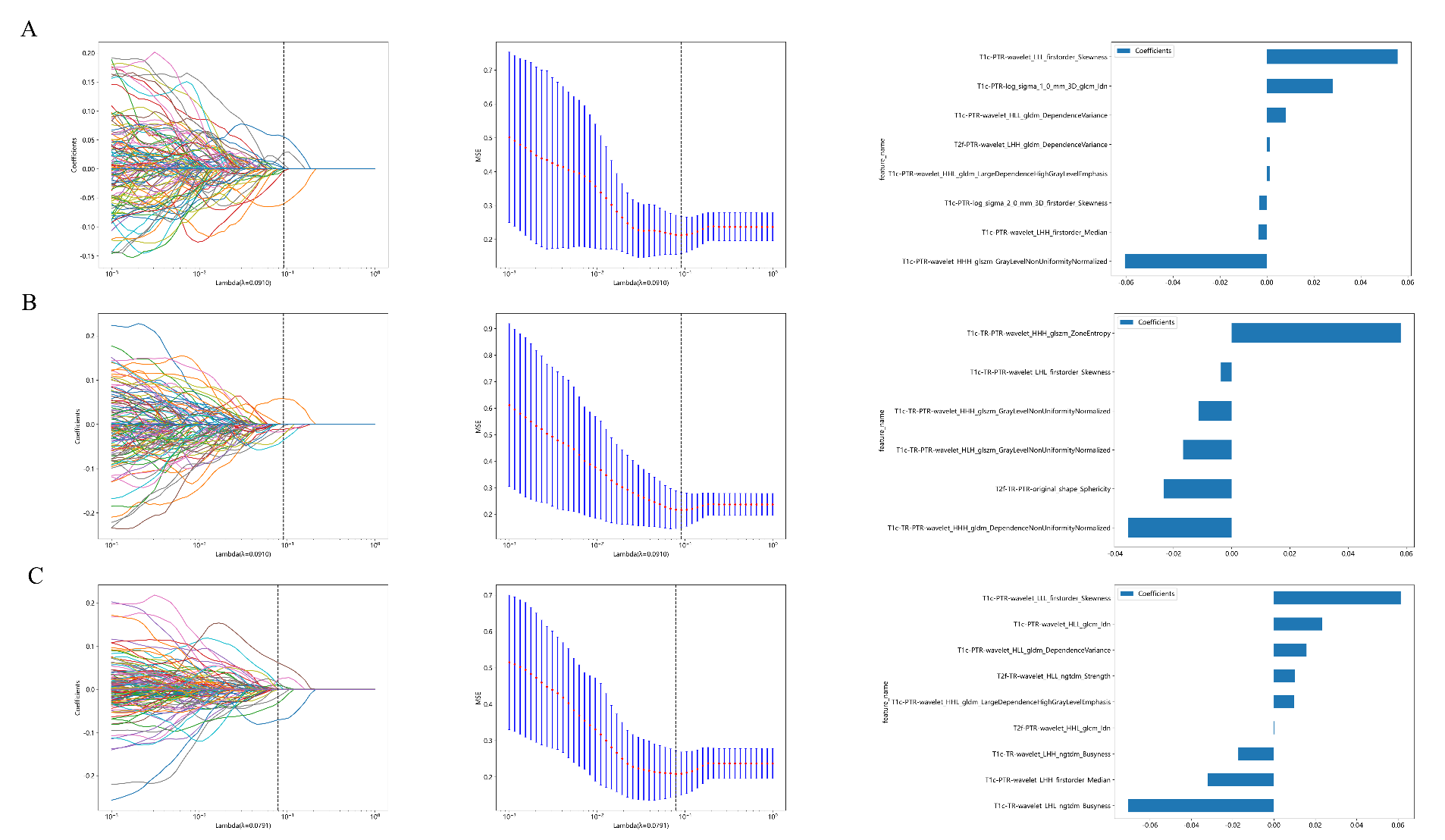


**Figure S3.** The intratumoral and peritumoral radiomics features dimension reduction by using a LASSO algorithm. The coefficients and MSE of 10 folds cross validation and the histogram of coefficients of PR (A), TPR_VOI-fusion_ (B), and TPR_feature-fusion_ (C) models respectively.


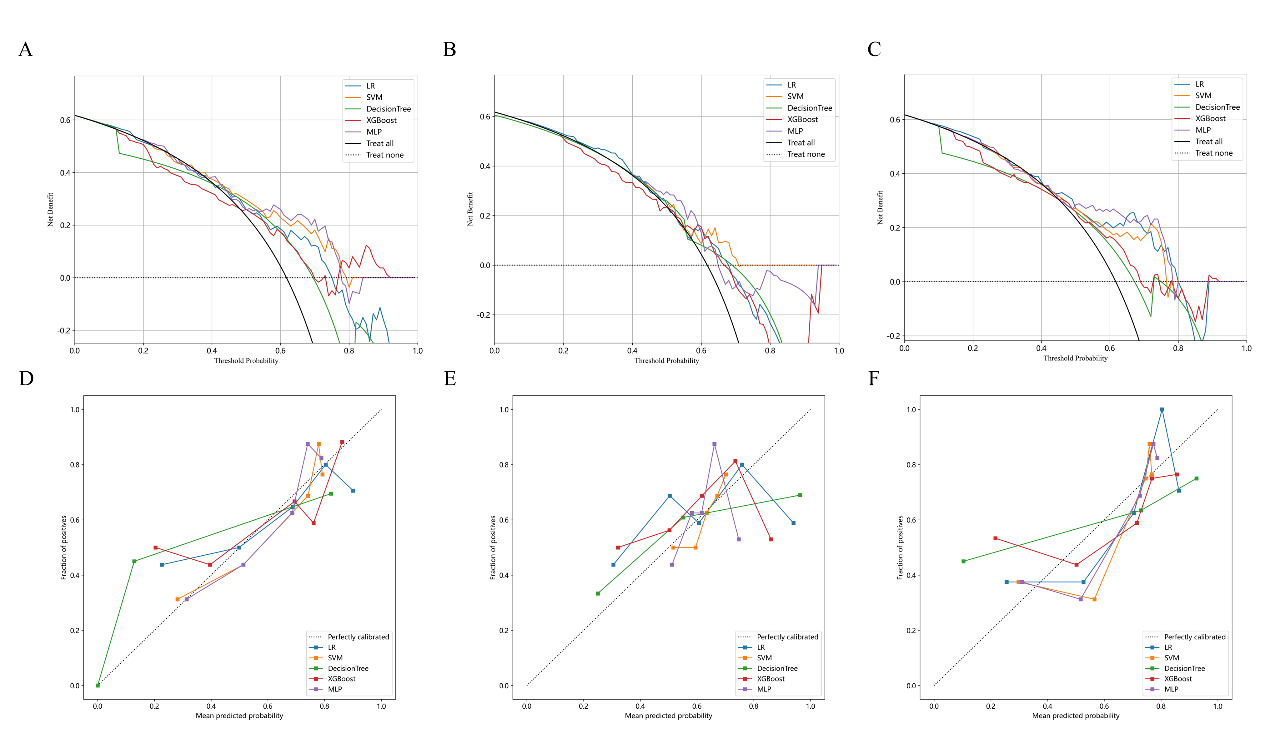


**Figure S4.** The DCA curves of TR_T1_ (A), TR_T2_ (B), and TR (C) models respectively. The calibration curves of TR_T1_ (D), TR_T2_ (E), and TR (F) models respectively.


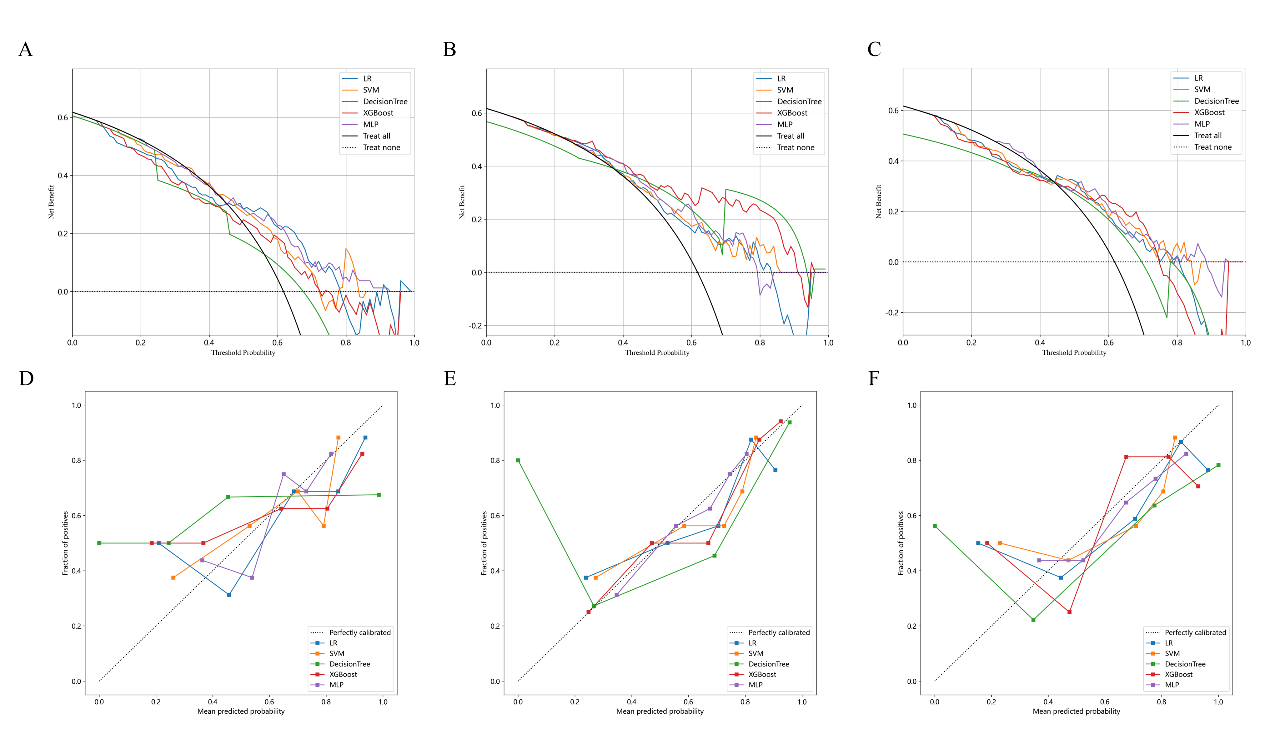


**Figure S5.** The DCA curves of PR (A), TPR_VOI-fusion_ (B), and TPR_feature-fusion_ (C) models respectively. The calibration curves of PR (D), TPR_VOI-fusion_ (E), and TPR_feature-fusion_ (F) models respectively.

**Table**

**Table S1** MRI acquisition parameters on 3.0T MRI scanner.

| Parameters | GE Healthcare Discovery MR750 | |
| --- | --- | --- |
| **CE-T1WI** | |  |
| TE/TR | | 24/1750 |
| Matrix | | 320×177 |
| **T2WI FLAIR** | |  |
| TE/TR | | 145/8400 |
| Matrix | | 512×512 |
| Slice thickness (mm) | | 5 |
| Slice gap (mm) | | 0.5 |
| Field of view (cm) | | 24-36 |
| No. of signal averages | | 1 |

**Table S2** Each evaluation index of TR_T1_, TR_T2_, and TR models of five machine learning classifiers in external test.

| Model | Classifier | AUC | 95%CI | Accuracy | Sensitivity | Specificity | PPV | NPV |
| --- | --- | --- | --- | --- | --- | --- | --- | --- |
| TR_T1_ | LR | 0.667 | 0.5389-0.7953 | 0.642 | 0.760 | 0.452 | 0.691 | 0.538 |
|  | SVM | 0.716 | 0.5953-0.8369 | 0.679 | 0.820 | 0.452 | 0.707 | 0.609 |
|  | DT | 0.586 | 0.4658-0.7058 | 0.667 | 0.820 | 0.419 | 0.695 | 0.591 |
|  | XGBoost | 0.638 | 0.5171-0.7597 | 0.642 | 0.740 | 0.484 | 0.698 | 0.536 |
|  | MLP | 0.733 | 0.6149-0.8510 | 0.642 | 0.800 | 0.387 | 0.678 | 0.545 |
| TR_T2_ | LR | 0.591 | 0.4536-0.7283 | 0.630 | 0.780 | 0.387 | 0.672 | 0.522 |
|  | SVM | 0.634 | 0.5091-0.7580 | 0.642 | 0.980 | 0.097 | 0.636 | 0.750 |
|  | DT | 0.555 | 0.4323-0.6781 | 0.642 | 0.960 | 0.129 | 0.640 | 0.667 |
|  | XGBoost | 0.535 | 0.3961-0.6742 | 0.617 | 0.760 | 0.387 | 0.667 | 0.500 |
|  | MLP | 0.590 | 0.4481-0.7312 | 0.679 | 1.000 | 0.161 | 0.658 | 1.000 |
| TR | LR | 0.701 | 0.5706-0.8307 | 0.654 | 0.800 | 0.419 | 0.690 | 0.565 |
|  | SVM | 0.722 | 0.6010-0.8429 | 0.654 | 0.820 | 0.387 | 0.683 | 0.571 |
|  | DT | 0.604 | 0.4817-0.7254 | 0.642 | 0.820 | 0.355 | 0.672 | 0.550 |
|  | XGBoost | 0.630 | 0.5026-0.7568 | 0.642 | 0.800 | 0.387 | 0.678 | 0.545 |
|  | MLP | 0.734 | 0.6149-0.8534 | 0.667 | 0.800 | 0.452 | 0.702 | 0.583 |
